# Therapeutic management and one-year outcomes in elderly patients with acute coronary syndrome

**DOI:** 10.18632/oncotarget.21260

**Published:** 2017-09-24

**Authors:** Esteban Orenes-Piñero, Juan M. Ruiz-Nodar, María Asunción Esteve-Pastor, Miriam Quintana-Giner, José Miguel Rivera-Caravaca, Andrea Veliz, Mariano Valdés, Manuel Macías, Vicente Pernias-Escrig, Nuria Vicente-Ibarra, Luna Carrillo, Miriam Sandín-Rollán, Elena Candela, Teresa Lozano, Francisco Marín

**Affiliations:** ^1^ Department of Cardiology, Hospital Clínico Universitario Virgen de la Arrixaca, (IMIB-Arrixaca), Universidad de Murcia, Murcia, Spain; ^2^ Department of Cardiology, Hospital General Universitario de Alicante, Alicante, Spain; ^3^ Department of Cardiology, Hospital General Universitario de Elche, Alicante, Spain

**Keywords:** acute coronary syndrome, elderly, antiplatelet therapy, revascularization, follow-up, Gerotarget

## Abstract

**Background:**

Elderly represents a subgroup of high-risk ACS patients due to their advanced age and other comorbidities. Unfortunately, they are also often under-represented in many studies and clinical trials. Furthermore, cardiologists commonly find difficulties in the choice of the antiplatelet treatment and even on whether invasive revascularization should be used. In this study, the management of elderly ACS patients regarding antiplatelet therapy and revascularization procedures will be analyzed.

**Methods:**

1717 ACS patients were consecutively included in this study from 3 tertiary Hospitals in the Southeast of Spain. Of them, 529 (30.8%) were ≥ 75 years. They were mainly male (60.7%) with a mean age of 81.4±4.7 years. Clinical characteristics, treatment received (antiaplatelet therapy, revascularization) and outcome were analyzed.

**Results:**

Regression analysis showed that being ≥ 75 years is independently associated with neither performing catheterization (79.6% *vs* 97.1%), nor revascularization (51.8% *vs* 72.5%), being the medical conservative treatment the election in these elderly patients (40.6% *vs* 18.9%) (*p* < 0.001 for all). Furthermore, ticagrelor prescription were significantly decreased in older patients (11.5% *vs* 19.6%; *p* < 0.001). Regarding patients outcome after one-year of follow-up, being ≥ 75 years was associated with death, major adverse cardiac events (MACE) and major bleeding (all of them *p* < 0.001). Importantly, nor performing catheterization was independently associated with MACE and death in Cox multivariate analysis in elderly patients.

**Conclusions:**

Elderly patients with ACS are undertreated both invasively and pharmacologically, and this fact might be associated with the observed worse outcomes.

## INTRODUCTION

Acute coronary syndromes (ACS) are the leading cause of death and high morbidity worldwide despite advances in pharmacological and interventional treatment, and are frequently caused by coronary atherosclerosis and myocardial ischemia [1, 2]. The main risk factor for ACS is age and its prevalence increases markedly as age increases. Thus, elderly patients represent a growing subgroup of ACS patients, and management of this population is particularly challenging [3]. Several studies have clearly shown that elderly patients present higher platelet reactivity under clopidogrel than younger patients [4]. In addition, this population has also a higher risk of bleeding events [5, 6] due several causes as renal or hepatic dysfunction, age-related decrease in weight, drug interactions with polypharmacy observed in these patients, anemia and other comorbidities [7].

Elderly patients are frequently under-represented in clinical trials, leading to uncertainty among clinicians about the relative efficacy and safety of some treatments in this group of patients and, as a consequence, they are less likely to receive evidence-based therapies [8]. A recent study found that in 80 ACS articles published from 2007 to 2009, only 13.8% of study participants were 75 years of age or older, and 29.7% of the trials contained exclusion criteria based on age [9]. According to the 2015 European Society of Cardiology (ESC) ACS guidelines, the recommended antiplatelet therapy consists of acetylsalicylic acid (ASA) in combination with one of the most potent P2Y12 inhibitors, ticagrelor or prasugrel, if no contraindication exists [10]. The American College of Cardiology/American Heart Association (ACC/AHA) guidelines, on the other hand, have no preference for clopidogrel, prasugrel or ticagrelor [11, 12]; however, it remains unclear on the choice of P2Y12 inhibitor in treating the elderly, although both guidelines emphasize the importance of considering the individual patient and a personalized therapy.

Because of the paucity and inconsistency of data on the use of newer antiplatelets and percutaneous intervention (PCI) in elderly in daily clinical practice, it is difficult for physicians to make well-grounded decisions in these patients. For that reason, the aim of this study is to analyze the management of elderly ACS patients regarding antiplatelet therapy and revascularization procedures.

## RESULTS

Clinical variables may be observed in Table 1. Elderly patients had more frequently previous stroke (15.9% *vs* 5.5%), peripheral artery disease (14.2% *vs* 6.6%), chronic kidney disease (48.5% *vs* 14.7%), anemia (44.4% *vs* 17.7%), coronary stenosis (34.2% *vs* 22.6%) and atrial fibrillation (18.2% *vs* 3.6%) when compared with younger patients (*p* < 0.001 for all comparisons).

**Table 1:** Demographic and clinical baseline characteristics of the patients included in this study

	< 75 years	≥ 75 years	p-value
**Age, n (%)**	1188 (69.2)	529 (30.8)	<0.001
**Male gender, n (%)**	904 (76.1)	321 (60.7)	<0.001
**Hypertension, n (%)**	719 (60.6)	440 (83.2)	<0.001
**Current smoker, n (%)**	572 (48.1)	62 (11.8)	<0.001
**Hyperlipidemia, n (%)**	693 (58.3)	332 (62.8)	0.111
**Diabetes, n (%)**	415 (34.9)	238 (45,0)	<0.001
**Atrial Fibrillation, n (%)**	43 (3.6)	96 (18.2)	<0.001
**Previous stroke, n (%)**	65 (5.5)	84 (15.9)	<0.001
**Peripheral artery disease, n (%)**	79 (6.6)	75 (14.2)	<0.001
**Coronary stenosis, n (%)**	269 (22.6)	182 (34.2)	<0.001
**Anemia, n (%)**	210 (17.7)	235 (44.4)	<0.001
**Chronic kidney disease, n (%)**	165 (14.7)	257 (48.5)	<0.001
**Previous ASA treatment, n (%)**	368 (31.0)	228 (43.1)	<0.001
**Previous clopidogrel treatment, n (%)**	129 (10.9)	99 (18.7)	<0.001
**Previous anticoagulant treatment, n (%)**	63 (5.3)	78 (14.7)	<0.001
**Previous coronary revascularization, n (%)**	265 (22.3)	160 (30.2)	0.001
**Reason for admission**			
**NSTEMI at hospitalization, n (%)**	473 (39.8)	277 (52.4)	<0.001
**STEMI at hospitalization, n (%)**	451 (38.0)	123 (23.3)	<0.001

Abbreviations: ACEI: Angiotensin converting enzyme inhibitor; ARB: Angiotensin receptor blocker; ASA: Acetylsalicylic acid; IADP: Adenosine diphosphate inhibitor.

As expected, older age is associated with a higher previous prescription of antithrombotic drugs, such as aspirin, clopidogrel or warfarin/acenocoumarol (43.1% *vs* 31.0%, 18.7% *vs* 10.9% and 14.7% *vs* 5.3%, respectively; *p* < 0.001 for all comparisons).

It is important to remark that elderly patients are medically under-treated at the moment of hospital admission as can be observed in Table 2. Elderly patients with an ACS are more frequently not treated with ASA loading dose when arriving at hospital Emergency Room compared with patients younger than 75 years (20.9 *vs* 32.5%; *p* < 0.001). Furthermore, other cardiovascular drugs such as β-blockers (77.9% *vs* 87.2%; *p* < 0.001), angiotensin converting enzyme inhibitors (ACEI) or angiotensin receptor blockers (ARB) (81.5% *vs* 87.7%; *p* = 0.001), were also under-administered in elderly during hospital stay; whereas no significant differences were found in statin administration.

**Table 2:** Pharmacological and percutaneous treatment during hospital stay and at discharge of patients included in this study

	< 75 years	≥ 75 years	p-value
**ASA load at admission**			
**None, n (%)**	248 (20.9)	172 (32.5)	<0.001
**300 mg, n (%)**	826 (69.5)	292 (55.2)	<0.001
**IADP during hospital stay**			
**None, n (%)**	48 (4.0)	45 (8.5)	<0.001
**Clopidogrel, n (%)**	818 (68.9)	421 (79.6)	0.001
**Prasugrel, n (%)**	89 (7.5)	2 (0.4)	<0.001
**Ticagrelor, n (%)**	233 (19.6)	61 (11.5)	<0.001
**β-blocker during hospital stay, n (%)**	1035 (87.2)	412 (77.9)	<0.001
**ACEI/ARB during hospital stay, n (%)**	1041 (87.7)	431 (81.5)	0.001
**Catheterism performed, n (%)**	1154 (97.1)	421 (79.6)	<0.001
**Medical conservative treatment, n (%)**	225 (18.9)	215 (40.6)	<0.001
**Percutaneous revascularization, n (%)**	861 (72.5)	274 (51.8)	<0.001
**ASA at discharge, n (%)**	1149 (96.7)	504 (95.3)	0.145
**IADP at discharge**			
**None, n (%)**	158 (13.3)	108 (20.4)	<0.001
**Clopidogrel, n (%)**	490 (41.2)	349 (66.0)	<0.001
**Prasugrel, n (%)**	193 (16.2)	1 (0.2)	<0.001
**Ticagrelor, n (%)**	347 (29.2)	71 (13.4)	<0.001
**Oral anticogulation at discharge, n (%)**	109 (9.1)	115 (21.7)	<0.001
**β-blocker at discharge, n (%)**	1003 (84.5)	408 (77.1)	<0.001
**ACEI/ARB at discharge, n (%)**	949 (79.9)	415 (78.4)	0.498

Abbreviations: ACEI: Angiotensin converting enzyme inhibitor; ARB: Angiotensin receptor blocker; ASA: Acetylsalicylic acid; IADP: Adenosine diphosphate inhibitor.

Interestingly, being older than 75 years is independently associated with less catheterization (79.6% *vs* 97.1%; *p* < 0.001) and revascularization (51.8% *vs* 72.5%; *p* = 0.001). Remarkably, the conservative approach was the most frequent one for patients ≥75 years (40.6% *vs* 18.9%; *p* < 0.001). Furthermore, ticagrelor and prasugrel administration at discharge significantly decreased in older patients (13.4 *vs* 29.2% and 0.2% *vs* 16.2%, respectively, *p* < 0.001 for both comparisons), whereas clopidogrel was more frequently administered (66.0% *vs* 41.2%; *p* < 0.001).

Remarkably, when analyzing the outcome of elderly patients depending on the antiplatelet therapy at discharge, it was observed that after one-year of follow-up, clopidogrel was associated with increased mortality (both, cardiac and non-cardiac) when comparing with ticagrelor (17.2% *vs* 5.6%, *p* = 0.008). In addition, the number of bleeding events according to the BARC (Bleeding Academic Research Consortium Definition of Bleeding) definition were higher in patients on clopidogrel when comparing with patients on ticagrelor (14.2% *vs* 5.6%, *p* = 0.034).

**Figure 1:**
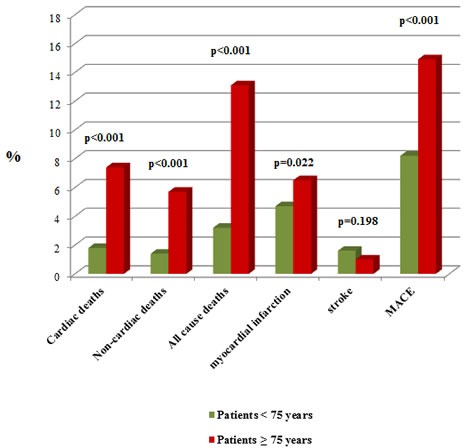
Patients’ outcome depending on their age Comparison of patients’ deaths and MACE after 1-year of follow-up.

**Figure 2:**
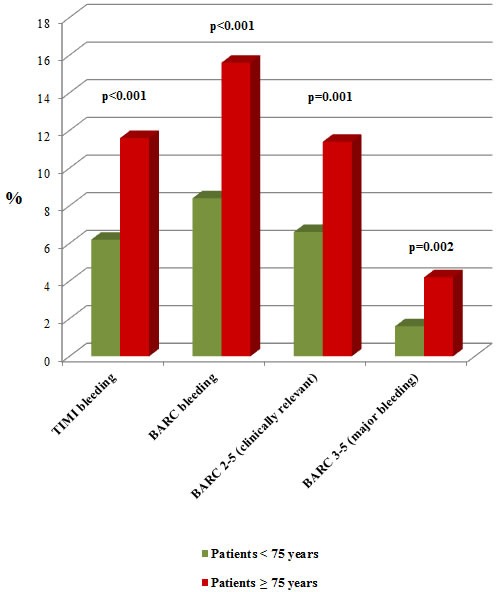
Patients’ outcome depending on their age Comparison of patients’ bleeding events after 1-year of follow-up.

Regarding one-year outcomes, significant differences in cardiac (7.4% *vs* 1.8%; *p* < 0.001) and non-cardiac deaths (5.7% *vs* 1.4%; *p* < 0.001) were observed for patients ≥75 years (Figure 1). In addition, MACE occurrence were also observed during follow-up (14.9% *vs* 8.2%; *p* < 0.001) and bleeding events were significantly higher using two different bleeding definitions, TIMI and BARC (11.6% *vs* 6.2%; 15.6% *vs* 8.4%, respectively) (*p* < 0.001 using both of them) when compared with younger patients (Figure 2).

**Figure 3:**
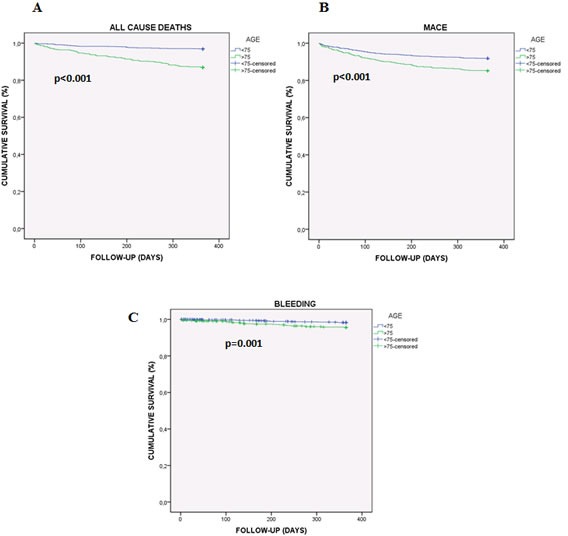
Kaplan-Meier cumulative survival curve showing the effect of being 75 or older on adverse events in ACS patients **A**. Effect of age on all cause deaths. **B**. Effect of age on MACE. **C**. Effect of age on bleeding events (BARC 3-5, major bleeding).

On the other hand, Cox analysis (Table 3) in patients older than 75 years showed that neither performing catheterization [HR: 2.97 (95% CI 1.89-4.66) *p* < 0.001] nor revascularization [HR: 2.09 (95% CI 1.33-3.28) *p* = 0.001] were associated with MACE in the univariate analysis. Moreover, left main coronary artery (LMCA) involvement [HR: 2.63 (95% CI 1.36-5.07) *p* = 0.004] and β-blockers at discharge [HR: 1.93 (95% CI 1.21-3.07) *p* = 0.006], were also associated. Additionally, when a multivariate analysis was carried out, non-performing catheterization [HR: 16.16 (95% CI 6.06-43.12) *p* < 0.001] and LMCA involvement [HR: 2.09 (95% CI 1.05-4.15) *p* = 0.036] continued independently associated with MACE. Regarding bleeding events, only taking clopidogrel at discharge was independently associated in both, univariate [HR: 2.67 (95% CI 0.97-7.41) *p* = 0.049] and multivariate analysis [HR: 2.92 (95% CI 1.05-8.14) *p* = 0.041]. Finally, neither performing catheterization [HR: 3.34 (95% CI 2.07-5.38) *p* < 0.001] nor revascularization [HR: 2.43 (95% CI 1.50-3.93) *p* < 0.001], LMCA involvement [HR: 2.15 (95% CI 1.02-4.53) *p* = 0.044], the use of non-pharmacological stents [HR: 2.47 (95% CI 1.15-5.34) *p* = 0.021] and clopidogrel prescription at discharge [HR: 2.77 (95% CI 1.00-7.66) *p* = 0.049] were associated with death in a Cox univariate analysis. However, in the multivariate, only non-performing catheterization remained as an independent variable associated with death [HR: 13.05 (95% CI 4.00-42.49) *p* < 0.001]. Furthermore, Kaplan-Meier method was performed to confirm that MACE and bleeding events were significantly associated with lower cumulative survival in elderly patients (Figure 3).

**Table 3:** Association of different clinical variables with MACEs, bleeding events and death during follow-up. Statistically significant values appear in bold

	MACEs		Bleeding events		Deaths	
	Univariate	Multivariate	Univariate	Multivariate	Univariate	Multivariate
	HR (95% CI) p	HR (95% CI) p	HR (95% CI) p	HR (95% CI) p	HR (95% CI) p	HR (95% CI) p
**Catheterism (non-performed)**	**2.97 (1.89-4.66) p<0.001**	**16.16 (6.06-43.12) p<0.0011**	1.06 (0.59-1.90) p=0.850		**3.34 (2.07-5.38) p<0.001**	3.32 (0.39-28.29) p=0.272
**LMCA involvement**	**2.63 (1.36-5.07) p=0.004**	**2.09 (1.05-4.15) p=0.036**	1.21 (0.52-2.83) p=0.655		**2.15 (1.02-4.53) p=0.044**	1.63 (0.46-5.75) p=0.445
**Revascularization (non-performed)**	**2.09 (1.33-3.28) p=0.001**	1.82 (0.93-3.54) p=0.080	1.50 (0.90-2.48) p=0.119	1.80 (0.95-3,38) p=0.070	2.45 (1.49-3.93) p<0.001	**13.05 (4.00-42.49) p<0.001**
**Significant lessions in CA**	0.19 (0.03-1.37) p=0.099	6.82 (0.88-52.83) p=0.066	6.47 (0.89-46.74) p=0.164		4,40 (0.60-32.05) p=0.164	
**Non-pharmacological stents**	0.71 (0.34-1.49) p=0.364		1.16 (0.60-2.25) p=0.655		2.47 (1.15-5.34) p=0.021	1.87 (0.84-4.19) p=0.126
**ASA at discharge**	1.07 (0.39-2.92) p=0.901		2.19 (0.95-5.06) p=0.067	2.19 (0.68-7.10) p=0.192	1.25 (0.46-3.44) p=0.661	
**Clopidogrel at discharge**	1.15 (0.73-1.83) p=0.544		**2.68 (0.97-7.41) p=0.049**	**2.92 (1.05-8.14) p=0.041**	2.77 (1.00-7.66) p=0.049	1.99 (0.56-6.99) p=0.286
**Anticoagulants at discharge**	1.28 (0.72-2.29) p=0.401		1.45 (0.86-2.46) p=0.166		1.32 (0.71-2.45) p=0.378	
**β-blockers at discharge**	**1.93 (1.21-3.07) p=0.006**	1.47 (0.74-2.92) p=0.269	1.42 (0.85-2.40) p=0.183		1.54 (0.92-2.57) p=0.099	1.54 (0.61-3.88) p=0.358
**ACEI and ARBs at discharge**	0.93 (0.54-1.61) p=0.799		1.10 (0.63-1.93) p=0.730		1.40 (0.83-2.37) p=0.213	
**Calcium antagonists at discharge**	1.37 (0.81-2.29) p=0.238		1.66 (0.83-3.35) p=0.155		1.52 (0.89-2.60) p=0.126	0.98 (0.32-2.93) p=0.964
**Statins at discharge**	0.85 (0.31-2.33) p=0.756		1.25 (0.51-3.12) p=0.626		0.97 (0.35-2.66) p=0.950	

## DISCUSSION

Management of ACS in elderly patients is a conflicting medical scenario because of the high prevalence of comorbidities and worse prognosis. Furthermore, the survival of patients with MIs decreases with patients’ age [14]. Given the prevalence and implications on survival in elderly patients, it is imperative that these patients receive the appropriate treatment regimens to maximize morbidity and mortality benefit; however, elderly patients are often under-represented in clinical trials and current ACS guidelines do not address specific changes for elderly management [10–12].

It is widely known that elderly have a variety of pharmacokinetic and pharmacodynamics changes that may alter treatment options, including changes in drug metabolism and clearance, as well as increased risk of side effects and interactions [15]. This fact can explain the under-administration of cardiovascular drugs such as ASA, β-blockers, ACEI or ARB during hospital stay and at discharge observed in our study. Nevertheless, the ACS guidelines recommend a daily maintenance dose of ASA among 81 mg to 325 mg in the absence of contraindications and without modification based on age, with a loading dose for ACS-PCI patients [10–12]. Historically, β-blockers have been under-used in the elderly. This may be a result of their side effects, such as fatigue and dizziness, or theories that they may be less effective in elderly patients because of a decrease in β-receptors [16]. However, ACS guidelines agree with the use of β-blockers, stating that they provide a greater benefit in elderly patients in preventing subsequent MI and death than in younger patients [10–12]. On the other hand, ACEI are commonly used in elderly patients as a relatively safe blood pressure medication, providing beneficial effects, such as reducing left ventricular remodeling post-MI [17]. ACS guidelines state that ACEI and ARB are beneficial in the elderly, particularly in heart failure or reduced left ventricular function and in reducing blood pressure, essential to avoid secondary events [10–12].

Interestingly, our study shows that being older than 75 years is independently associated with neither performing catheterization nor revascularization, being the medical conservative treatment the election for patients older than 75 years. Importantly, invasive procedures are increasing in elderly populations with time and this could lead us to better outcomes; however, as highlighted in this study, we are still far from an optimum treatment in these patients. There might be several reasons for this decrease in interventional treatment for elderly patients. Among them, can be highlighted: (i) the higher incidence of comorbidities, including more extensive atherosclerosis, hypertension and renal insufficiency, as well as their more frequent presentation with hemodynamic instability; (ii) the higher prevalence of calcified lesions, tortuous lesions, ostial lesions, multi-vessel disease and left main stenosis; and (iii) bleeding, causing harmful effects through hypovolemia, hypotension, reduced oxygen carrying capacity, drug discontinuation and blood transfusion [18]. Despite the decrease in PCI observed in this study, the benefits of interventional revascularization have been demonstrated elsewhere.

Recently, a large multinational registry revealed no difference in death, stroke or MI among octogenarians revascularized with PCI *versus* CABG surgery at a median follow-up of 1088 days [19]. Moreover, a systematic review of clinical studies performed to identify the health-related quality of life (HRQOL) after PCI in the elderly, showed that they have significant improvements in cardiovascular well-being after PCI [20]. Another recent study conducted in octogenarians, also exhibited the benefits of PCI in a cohort of 353 consecutive patients with ACS [21]. In overall cohort, 5-years all-cause mortality was 46.2% *vs* 89.5% comparing PCI and non-PCI groups, respectively. In addition, a statistically significant association between PCI and reduced long-term mortality was found. Furthermore, a significant improved mean survival rates was observed for PCI-treated patients [21]. All these consistent data, together with the result of the Cox univariate and multivariate analysis performed in the present study, confirm that performing catheterization and revascularization have better outcomes than conservative medical therapy in elderly presenting ACS.

Another intriguing result of this study is that ticagrelor and prasugrel prescription during hospital stay and at discharge was less frequently in older patients, whereas clopidogrel administration increased. Current guidelines recommend tailoring antithrombotic treatment according to body weight and renal function in the elderly; however, it remains unclear on the choice of P2Y12 inhibitor. The TRITON-TIMI 38 trial showed that prasugrel intake was associated with a 32% increased risk of bleeding especially in the elderly. Hence, prasugrel is generally not recommended in patients ≥ 75 years [22]. On the other hand, the PLATO trial showed a greater absolute (2.8% *vs* 1.3%) and relative reduction (17.0% *vs* 15.0%) of ischemic end-points in elderly compared to younger patients, with lower incidence the primary composite end-point of cardiovascular death, MI or stroke when comparing ticagrelor *vs* clopidogrel (9.0 *vs* 10.7%; *p* = 0.0025) [23]. Additionaly, there was no difference between clopidogrel and ticagrelor groups in the rates of total major bleeding or severe bleeding [23]. This trial, therefore, concluded that ticagrelor may be a better option than clopidogrel for patients with ACS for whom an early invasive strategy with PCI is planned. The results of the our study confirm that ticagrelor administration was associated with a decrease in total deaths (cardiac and non-cardiac) and bleeding events according to the BARC definition after one year of follow-up, thus highlighting the importance of an adequate medical treatment in elderly. However, this latter observation should be taken with caution because patients on ticagrelor at discharge had less comorbidities, such as AF (0.7% *vs* 10.5%; *p* < 0.001), anemia (15.6% *vs* 29.3%; *p* < 0.001), diabetes (26.6% *vs* 41.7%; *p* < 0.001), previous ischemic disease (4.8% *vs* 9.9%; *p* = 0.002), peripheral artery disease (3.3% *vs* 10.8%; *p* < 0.001) and chronic kidney disease (14.6% *vs* 28.6%; *p* < 0.001), when compared with patients on clopidogrel, thus biasing in better outcomes in patients on ticagrelor during the follow-up.

## MATERIALS AND METHODS

### Patients

The ACHILLES registry is a prospective, multicentric study carried out in three tertiary hospitals located in the Southeast of Spain with catheterization facilities, including the existence of a PCI program. 1717 ACS patients were consecutive included with a definitive diagnosis of ACS between February 2014 and December 2015 and treated according to their physician criteria. Of them, 532 (30.9% were 75 years of age or older). They were mainly male (60.7%) with a mean age of 81.4±4.7 years, as can be observed in Table 1. The study was approved by the Clinical Research Ethics Committees of each hospital and the signing of the consent was necessary for all patients included in the study. An external audit of the registry data by an independent CRO was conducted in all participating hospitals. The correct inclusion of patients, the analysis of the available data and the possible existence of patients not included during the recruitment period was investigated.

### Variables and definitions

Demographic, clinical, therapeutic and follow-up data of all patients were collected. Demographic data included age, gender, weight, height and body mass index (BMI). Clinical data comprised cardiovascular risk factors and comorbidities, among others. Therapeutic data included use of antiplatelets (aspirin and P2Y12 inhibitors, including initial oral loading dose and switching when necessary) and anticoagulants at the time of admission and during hospital stay. In addition, treatment during stay and at discharge was also recorded. In patients undergoing cardiac catheterization, procedure details were also recorded (moment of the catheterization, access site, number of diseased vessels, type of revascularization and number/size of stents implanted).

For bleeding risk assessment and ischemic risk stratification, the Crusade bleeding score (http://www.crusadebleedingscore.org), and the GRACE score (http://www.gracescore.org) were calculated, as they are the most discriminatory for major bleeding and ischemic risk in ACS patients undergoing coronary angiography, respectively [10]. Definition of bleeding categories was made according Bleeding Academic Research Consortium (BARC) criteria. We also collected major TIMI bleeding episodes. The BARC consensus document proposed a hierarchical grading system to classify bleeding events in cardiovascular investigations standardizing key ischemic end-point definitions in studies aimed at evaluating coronary stents [13]. For this analysis, all bleeding events were classified according to the BARC hierarchical bleeding scale by a clinical event committee adjudication using data prospectively collected. Bleeding events were divided by the committee according to three main groups: total bleeding (categories 1-5), clinically relevant (categories 2-5) and major bleeding (categories 3-5). TIMI major bleedings were also recorded: any intracranial bleeding, clinically overt signs of hemorrhage associated with a drop in hemoglobin of > 5 g/dL, or fatal bleeding.

Follow-up was performed prospectively by telephone contact by qualified personal with each patient at 3, 9 and 12 months after discharge. Medical records were comprehensively reviewed to check details about any event or changes in antiplatelet medication. Bleeding events and MACEs were recorded as outcome variables. MACEs included cardiac death, any type of ACS and stroke.

### Statistical analysis

Continuous variables were tested for normal distribution by the Kolmogorov-Smirnov test. The normal distributed continuous variables are shown as mean ± standard deviation, and those non-parametrically distributed are shown as median (interquartile range). Categorical variables are presented as frequencies (percentages). Comparisons of the groups for continuous variables were performed with the unpaired *t*-test for independent samples or the Mann-Whitney U-test (as appropriate). The comparison of discrete variables was performed by chi-square test or Fisher test (as appropriate). Correlation between two continuous variables was performed using the Pearson or Spearman test (as appropriate). Multivariate analyses were carried out to test the predictive discrimination of several demographic, clinical and therapeutic variables in association with bleeding events and MACE. The overall free survival rates were calculated using the Kaplan-Meier method, and differences determined using the log-rank test. The effect on prognosis was calculated for MACE, bleeding risk and death by using a Cox proportional hazards regression. Only those variables showing values with *p* < 0.150 in the univariate analysis were incorporated into the multivariate model. A p value of less than 0.05 was considered to be significant. The statistical analyses were carried out using the Statistical Package for the Social Sciences (SPSS), version 21.0 for Windows software program (Chicago, Illinois, USA).

## STUDY LIMITATIONS

This study has several limitations. Although our study was conducted in three different hospitals, most of patients were Caucasians, and the data could not be extrapolated to other ethnic groups. Moreover, the fact that the participating hospitals had catheterization facilities, including the existence of a PCI program, could be related to more invasive hospital management and this should be taken into account when interpreting the results of the study [24]. In addition, patients should be discharged with an ACS diagnosis as an inclusion criterion, so those who died during hospital stay were lost. Additionally, we have not recorded the number of deaths during hospital stay. Furthermore, although follow-up of novel antiplatelet administration was performed prospectively by telephone contact at 3, 9 and 12 months after discharge and medical records were comprehensively reviewed to check details about any event or changes in antiplatelet medication, data should be carefully considered. Despite these limitations, the willingness of the registry guarantees a very high quality of the data that has been corroborated by an external and independent audit.

## CONCLUSIONS

This paper shows that ACS patients over 75 years old are undertreated both invasively and pharmacologically, being the medical conservative treatment the elective one. Importantly, this decrease in more aggressive treatments due to comorbidities and bleeding in the elderly, contrary to the current ACS European and American guidelines, could participate in the observed worse outcomes.
